# Deep-Learning-Based Analysis of Electronic Skin Sensing Data

**DOI:** 10.3390/s25051615

**Published:** 2025-03-06

**Authors:** Yuchen Guo, Xidi Sun, Lulu Li, Yi Shi, Wen Cheng, Lijia Pan

**Affiliations:** Collaborative Innovation Center of Advanced Microstructures, School of Electronic Science and Engineering, Nanjing University, Nanjing 210093, China; 201240031@smail.nju.edu.cn (Y.G.); dg21230047@smail.nju.edu.cn (X.S.); 502024230029@smail.nju.edu.cn (L.L.)

**Keywords:** deep learning, electronic skin, data processing, health monitoring, human–machine interaction

## Abstract

E-skin is an integrated electronic system that can mimic the perceptual ability of human skin. Traditional analysis methods struggle to handle complex e-skin data, which include time series and multiple patterns, especially when dealing with intricate signals and real-time responses. Recently, deep learning techniques, such as the convolutional neural network, recurrent neural network, and transformer methods, provide effective solutions that can automatically extract data features and recognize patterns, significantly improving the analysis of e-skin data. Deep learning is not only capable of handling multimodal data but can also provide real-time response and personalized predictions in dynamic environments. Nevertheless, problems such as insufficient data annotation and high demand for computational resources still limit the application of e-skin. Optimizing deep learning algorithms, improving computational efficiency, and exploring hardware–algorithm co-designing will be the key to future development. This review aims to present the deep learning techniques applied in e-skin and provide inspiration for subsequent researchers. We first summarize the sources and characteristics of e-skin data and review the deep learning models applicable to e-skin data and their applications in data analysis. Additionally, we discuss the use of deep learning in e-skin, particularly in health monitoring and human–machine interactions, and we explore the current challenges and future development directions.

## 1. Introduction

Electronic skin is an integrated flexible electronic system that mimics the sensory functions of human skin. Compared with conventional rigid devices, electronic skin has better breathability and flexibility and can realize a seamless fit with the skin, reducing the discomfort of people wearing it [1]. In addition, similar to human skin, it is self-healing and biocompatible. Highly integrated e-skin can detect multimodal signals and, through wireless communication, enable the stable and continuous measurement of multiple signals for a long time. This makes it widely used in the field of health monitoring [2,3,4,5], human–computer interactions [6,7,8,9], and other areas. By monitoring physiological parameters, such as heart rate, blood oxygen, muscle activity, etc., e-skin can detect and intervene in potential health problems at an early stage or provide health coaches with data for providing scientific training methods. E-skin can also accurately sense temperature, tactile signals, and other perceptions of the external environment, which is key for developing robots with diverse capabilities. This enables complex real-world applications like human–machine emotional interactions and gesture recognition [10,11].

The realization of the practical functions of e-skin is highly dependent on the efficient processing and interpretation of complex sensing data. The sensing data of electronic skin are typically high-dimensional and temporal in nature, but they are often plagued by noise and artifacts [12,13]. Nowadays, commonly used data analysis methods, such as those based on statistical and frequency domain analysis, can efficiently process single-modal or linear signals [14], but their classification accuracies are generally lower than 80% in multimodal fusion and dynamic environments (e.g., the traditional machine learning methods KNN and SVM are only 79% and 70% accurate in complex gesture recognition [10]). Moreover, traditional methods rely on manual feature engineering, which is difficult to adapt to dynamic environments such as changes in wearing position [15].

**Figure 1:**
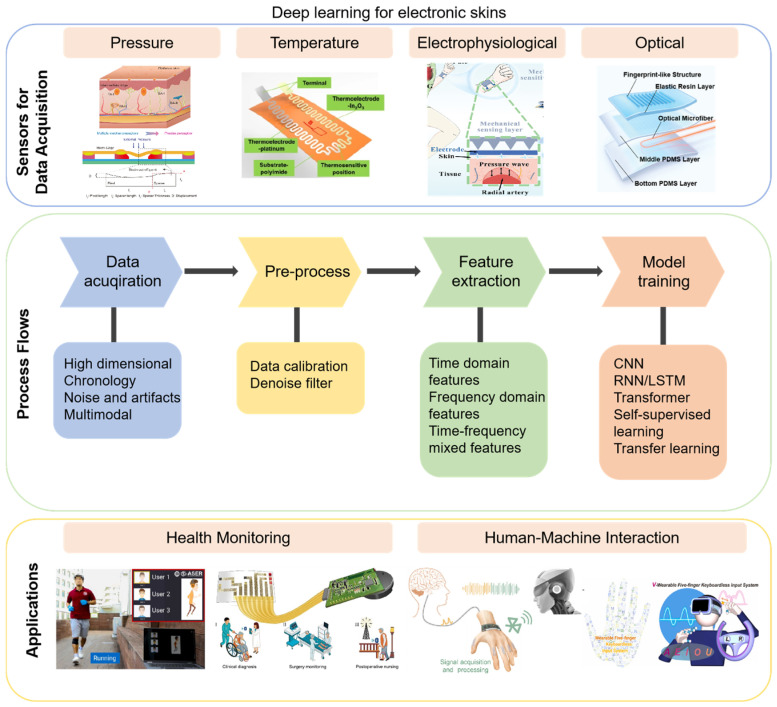
Overview diagram of the sensors, process flows, and applications of deep-learning-based e-skin. Image representing pressure was reproduced with permission from ref [30]. Copyright 2023, Springer Nature. Image representing temperature was reproduced with permission from ref [31]. Copyright 2021, Springer Nature. Image representing electrophysiological was reproduced with permission from ref [32]. Copyright 2023, John Wiley and Sons. Image representing electrophysiological was reproduced with permission from ref [33]. Copyright 2021, John Wiley and Sons. Left image representing health monitoring was reproduced with permission from ref [34]. Copyright 2023, Elsevier. Right image representing health monitoring was reproduced with permission from ref [35]. Copyright 2022, John Wiley and Sons. Left image representing human–machine interaction was reproduced with permission from ref [10]. Copyright 2024, John Wiley and Sons. Right image representing human–machine interaction was reproduced with permission from ref [36]. Copyright 2022, Elsevier.

With the rapid development of artificial intelligence technology, deep-learning-based algorithms provide an effective solution for complex data analysis. Deep learning breaks through the bottleneck of traditional methods through hierarchical feature abstraction, the core of which lies in the ability of the multilayer neural network structure to automatically learn hierarchical feature representations in data. Convolutional neural networks (CNNs) can automatically extract spatially distributed features of sensor arrays by exploiting the properties of local connectivity and weight sharing [16], and long short-term memory networks (LSTMs) can effectively model the temporal dependence of bioelectrical signals through their unique gating mechanism [17]. These deep learning models benefit from backpropagation algorithms and gradient descent optimization methods to extract features and recognize patterns from complex data without human intervention. In addition, the popularity of deep learning frameworks such as TensorFlow and PyTorch (version 2.5) has made model training and deployment easier. This flexibility allows deep learning to effectively process multimodal data from sensors such as pressure, temperature, and bioelectricity, providing more accurate analysis and prediction for a variety of application scenarios (Figure 1) [18,19,20]. In addition, deep-learning-based methods also assist electronic skin in adapting to dynamic environments more efficiently, supporting real-time responses and personalized prediction. For example, Yang et al. achieved an average accuracy of 91% in laryngeal motion speech recognition using the AlexNet model, representing a 15% improvement over traditional methods [21]. In addition, a graph neural network (GNN) was successfully applied to model the topology of flexible sensor networks, significantly improving the robustness of human pose reconstruction [22].

Deep learning has significantly enhanced the capabilities of e-skin in multimodal data processing and pattern recognition, but its practical applications still face several challenges. First, the lack of high-quality labeled data restricts the model generalization ability. Existing datasets are mostly confined to laboratory environments with small sample sizes, which cannot meet the requirements of tens of thousands of human-scale samples needed for clinical-grade applications [23]. Researchers are working on the construction of multimodal and large-scale datasets, and they hope to enhance the robustness of these models by simulating diverse physiological data in real-life scenarios. In addition, techniques such as transfer learning and reinforcement learning can be used to effectively utilize the limited labeled data and improve the adaptability of the model in different tasks [24,25]. Second, the contradiction between model computational intensity and device power consumption also restricts the application of these models, especially on embedded platforms. The inference latency of traditional deep learning models such as ResNet-50 can reach hundreds of milliseconds, making it difficult to meet real-time interaction needs. To address this challenge, two optimization schemes have been proposed in academia. At the algorithmic level, optimization algorithms such as knowledge distillation and model compression (e.g., quantization and pruning) are used, which can reduce model parameters while maintaining high accuracy [26]. At the hardware level, the use of an integrated memory-computing chip reduces energy consumption and improves the energy efficiency ratio [27]. Furthermore, the lack of personalized adaptation mechanisms leads to unstable model performance across individuals [28]. To this end, federated learning solves the problem of multicenter data collaboration through distributed training and privacy-preserving techniques while improving model robustness across individuals through personalized modeling optimization algorithms (e.g., FedProx) [29]. These issues limit the widespread application of e-skin in fields such as health monitoring and robotic interaction. Based on the above problems, this review comprehensively introduces deep learning analysis techniques for electronic skin sensing data, aiming to provide a reference for the integration of artificial intelligence and electronic skin.

In this paper, we first introduce the data sources of e-skin sensors and analyze the unique characteristics of these data. Next, several commonly used deep learning models and methods are introduced, and the key steps of data preprocessing and feature extraction are analyzed in depth. Then, we summarize the research progress of e-skin in the fields of health monitoring and human–machine interaction in recent years and discuss its innovations and achievements in practical applications. Finally, the opportunities and challenges faced by deep-learning-based e-skin and possible solutions are discussed.

## 2. Data Sources of Electronic Skin

The primary function of e-skin lies in the effective sensing and collection of various types of data, which is enabled by a variety of sensor types. By capturing physical, biological, and chemical signals, these sensors provide rich data for deep learning models, thus supporting the widespread application of e-skins in fields such as healthcare [37,38], robotics [39,40], and virtual reality [41,42]. In the following, we will take a detailed look at the common flexible sensors of pressure and temperature and electrophysiological and optical sensors and explore their characteristics in data acquisition as well as their relationship with deep learning analysis.

### 2.1. Pressure Sensors: Decoding Tactile Patterns

**Figure 2:**
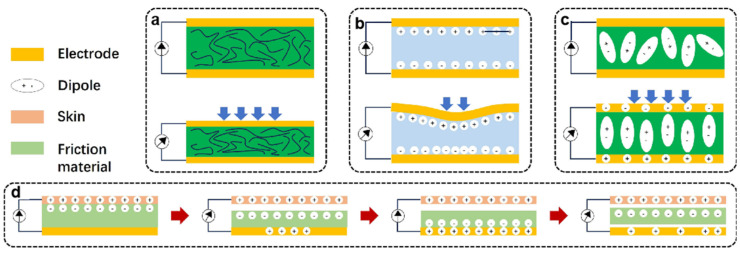
Schematic diagram of common pressure sensing mechanisms [57]. (**a**) Resistive, (**b**) capacitive, (**c**) piezoelectric, (**d**) triboelectric. Copyright 2024, OAE Publishing Inc.

Pressure sensors are one of the core functions of e-skins and are mainly classified into four categories: resistive, capacitive, piezoelectric, and triboelectric sensors [43,44,45,46] (Figure 2). Both resistive and capacitive sensors use changes in resistance or capacitance values caused by the deformation of the material due to the action of external mechanical forces for sensing. Resistive sensors usually consist of three parts: intrinsically conductive elastomeric materials or composites of conductive materials with elastomers and stretchable materials [47]. Capacitive sensors usually consist of a pair of electrodes and a dielectric layer sandwiched between the electrodes. Conductive materials such as metal films, metal nanowires, graphene, carbon black, and carbon nanotubes have been widely used to prepare electrodes for capacitive electronic skin. Their dielectric layers usually consist of elastic dielectric materials with micro/nanostructures. The microstructures can greatly enhance the sensitivity of these two types of sensors. Zhang et al. developed ultrathin, ultralight, and gas-permeable versatile electrospun micropyramid arrays through a self-assembly technology based on wet heterostructured electrified jets [48]. The capacitive sensor based on this microstructure has high sensitivity (19 kPa^−1^), an ultralow detection limit (0.05 Pa), and an ultrafast response (≤0.8 ms).

Piezoelectric and triboelectric sensors are relatively new sensing methods. Unlike traditional resistive and capacitive sensors, these two sensing methods offer faster response times and higher output power. Piezoelectric sensors produce a change in piezoelectric potential on the surface of the material due to an externally applied mechanical force that causes a change in the dipole deflection or dipole moment of the material. Triboelectric sensors, on the other hand, are based on the coupled effects of contact initiation and electrostatic induction and utilize external mechanical force to cause an electrode potential difference for sensing. Piezoelectric sensors must be fabricated from piezoelectric ceramics (e.g., barium titanate, lead zirconate titanate, and sodium and potassium niobates [49,50,51]) or polymers (e.g., polyvinylidene fluoride (PVDF), its copolymers poly(vinylidene fluoride-trifluoroethylene) (P(VDF-TrFE)), and poly(vinylidene fluoride-hexafluoropropylene) (P(VDF-HFP)) [52,53,54])), while triboelectric sensors do not have this material restriction. Both solids, liquids, and gases produce a friction point effect when in contact with each other and are capable of being used as electrostatic sensing devices.

Of course, a single sensor or sensing mode cannot meet the demands of large-scale sensing tasks and basic applications. Arrays of pressure sensors in e-skin can perceive tactile information such as contact strength, distribution, and dynamic changes. The data from these sensors are presented as a two-dimensional matrix with high resolution. Sundaram et al. developed a scalable all-haptic glove [55]. A large-scale haptic dataset containing 135,000 frames was obtained by collecting haptic maps generated by 548 pressure sensors on the glove under grasping actions. The convolutional neural network linked the temporal and spatial relationships between the haptic signals and successfully recognized 26 object grasping patterns. This joint physical–virtual optimization framework significantly improves the spatio-temporal resolution of haptic feedback. More complex designs of pressure sensors introduce multilayer structures or flexible material optimization. Using a new composite of elastic polymers and metal particles, Guo et al. proposed self-healing artificial innervation foam piezoresistive tactile sensors [56]. They are capable of measuring both vertical pressure and shear direction simultaneously. These high-dimensional data provide rich inputs for deep learning models, facilitating human–machine interaction in augmented reality and robotic skin applications. In addition, this type of sensing data can be combined with other modalities (such as temperature or chemical signals) to support more complex object recognition or dynamic interactions.

### 2.2. Temperature Sensors: Evaluating Thermal Dynamics and Environmental Characterization

Temperature sensing is one of the core functions of e-skin, which is able to mimic the ability of human skin to sense temperature changes. Temperature sensors in e-skin are usually based on the thermoelectric effect or the changes in the electrical properties of temperature-sensitive materials. Common principles of temperature sensing include thermoelectric effects, resistance changes, thermistors, and changes in the properties of semiconductor materials [58,59]. These sensors can detect changes in ambient temperature, body temperature, the presence of heat sources, and differences in temperature distribution. Temperature sensing is one of the core functions of e-skin, which is able to mimic the ability of human skin to sense temperature changes. Temperature sensors in electronic skin are usually based on the thermoelectric effect or electrical changes in temperature-sensitive materials. Common temperature sensing principles include the thermoelectric effect, resistance changes, thermistors, and semiconductor material property changes [11,12]. They can detect changes in ambient temperature, body temperature, the presence of heat sources, and differences in temperature distribution. Resistive temperature sensors are the predominant mechanism of operation. The heat transfer/dissipation mechanism or geometry of a thermistor can change significantly with temperature [57]. Common thermistor materials include metal oxide and silicon nitride [60]. In addition, organic hydrogels with a high ionic concentration and ionic mobility have an increase in conductivity in response to an increase in temperature. Ionic hydrogels are also widely used in temperature sensors [61]. The signals output from these sensors are mostly continuous time series data and show strong correlation when combined with pressure sensors. For example, highly integrated temperature sensors on robotic hands can more accurately determine the temperature of objects, the direction of fluid flow, and the temperature distribution on curved surfaces by simultaneously analyzing the temperature and pressure distribution at contact points [62,63].

**Figure 3:**
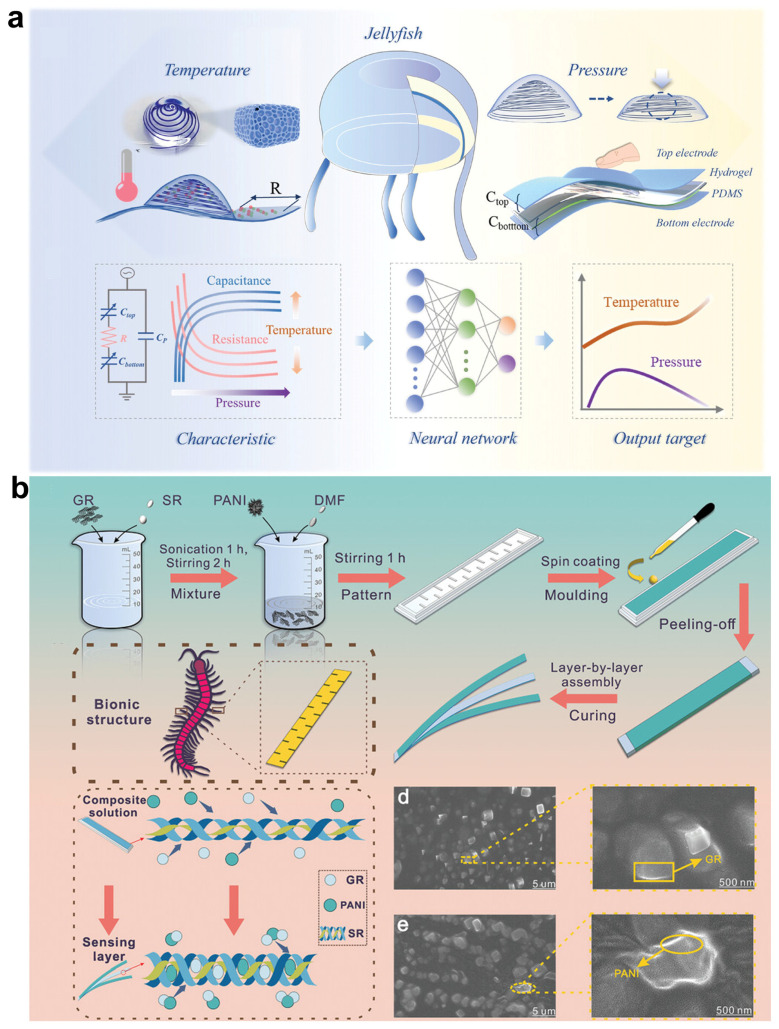
Temperature sensors based on bionic design. (**a**) Jellyfish-inspired sensor device schematic. Machine learning can be used to decouple temperature and pressure by analyzing capacitance and resistance signals under different conditions [64]. Copyright 2024, John Wiley and Sons. (**b**) Flowchart of DMSTS preparation based on centipede’s foot and schematic diagram of DMSTS bionic structure sensing layer [65]. Copyright 2024, John Wiley and Sons.

Biomimetic design has also been widely applied in the fabrication of temperature sensors. Inspired by the ‘mesoglea’ and ‘ectoderm’ structures of jellyfish, researchers have combined hydrogels with flexible frameworks to design a highly sensitive temperature and pressure sensor with a structure similar to the umbrella of jellyfish [64]. In this design, capacitance and resistance to pressure and temperature differ significantly in sensitivity and trends. The authors successfully used a neural network based on the Keras sequential framework to decouple the data and achieve accurate simultaneous measurements of temperature and pressure. This design significantly improves the pressure sensitivity and effectively reduces the signal drift, achieving a linear temperature sensitivity of −1.64 °C^−1^ (Figure 3a). In addition, researchers implemented a novel flexible dual-mode strain/temperature sensor (DMSTS) using graphite powder/polyaniline/silicone rubber composites that mimic the bionic microstructure of a centipede’s foot [65]. The DMSTS possesses an excellent strain range of 177% and a low detection limit of 0.5% strain, with a high sensitivity of 10.3 below 90 °C. Due to the photothermal properties of graphite powder and polyaniline, the DMSTS has broad application prospects in human motion detection, infrared imaging, and photothermal effects. When integrated into smart sensing systems, it enables dynamic non-contact temperature measurement, the detection of human micro-expressions, and monitoring of joint movements (Figure 3b). The data generated by these optimized temperature sensors have higher accuracy, providing strong support for subsequent deep learning models, making temperature sensing more accurate and reliable in a variety of application scenarios.

### 2.3. Electrophysiological Sensors: Interpreting the Dynamic Patterns of Physiological Signals

Electrophysiological signals are electrical signals generated by biological activities within the body, and long-term monitoring of bioelectric signals on the skin plays a crucial role in human–machine collaboration, disease prevention, and precise diagnosis [66]. Due to the need for close contact with the skin, good adhesion and low contact impedance between the sensor’s electrodes and the skin are required. Conventional Ag/AgCl gel electrodes possess good signal quality, but they are relatively hard, and the gel dehydrates with prolonged use, degrading the signal quality and causing skin irritation. In contrast, biocompatible conductors such as gold [67], conductive polymers [68], graphene [69,70], or nanowires [71] are often chosen as ultrathin conductors. By attaching electrodes to specific locations on the human body, electrophysiological signals such as electrocardiograms (ECG), electroencephalograms (EEG), electromyograms (EMG), and surface electromyograms (sEMG) can be measured [72,73,74]. These data usually appear in the form of time series with complex dynamic properties such as non-stationarity, strong noise, and nonlinear relationships between signals.

Moin et al. proposed the Hyperdimensional Computing (HDC) architecture, where raw data or preprocessed features of the finger sEMG are projected onto 1000D bipolar ((−1, +1)1000, for simplicity in describing mathematical operations) or binary ((0, 1)1000, for implementation with digital logic) hypervectors with information fully distributed across all bits, analogous to the way the human brain utilizes vast circuits of neurons and synapses for learning and recall [75]. The framework includes in-sensor training, inference in multiple new contexts, incremental updates to adapt to new contexts, and improved accuracy. It is able to perform real-time inference for gesture classification with a latency of 50 ms.

**Figure 4:**
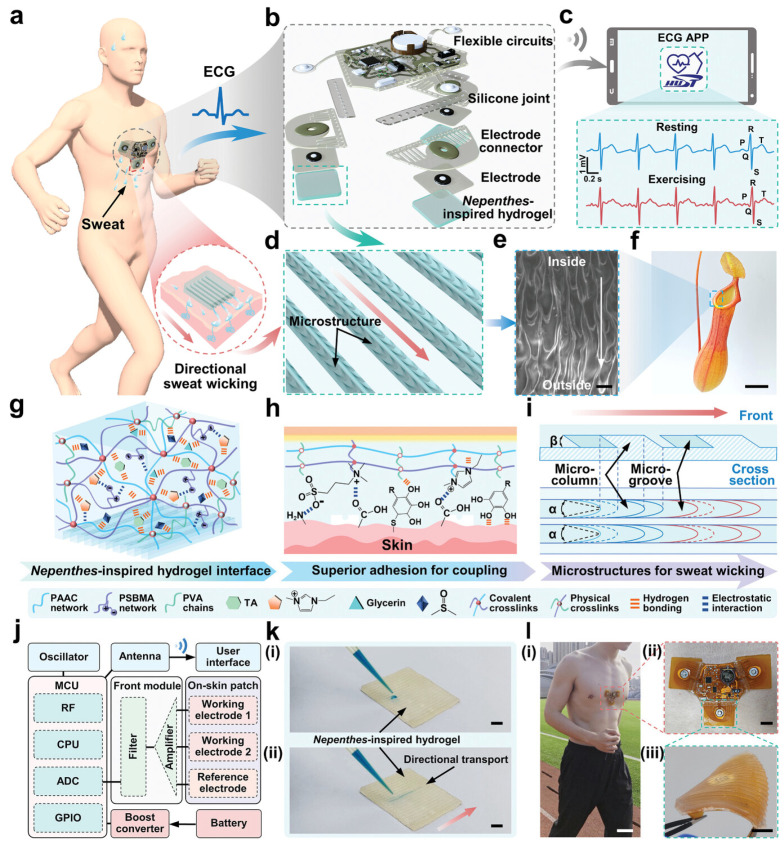
Design of the nepenthes-inspired hydrogel hybrid system [74]. (**a**) Schematic of the hydrogel system on human skin for ECG recording, with inset showing sweat-wicking NIH layer. (**b**) Exploded 3D model of the NIH hybrid system. (**c**) ECG signals from resting and exercising states displayed on the app. (**d**) Nepenthes-inspired microstructures of the hydrogel interface. (**e**,**f**) SEM image (**e**) and photograph (**f**) of nepenthes lip. (**g**) NIH network composition schematic. (**h**) Hydrogel/skin adhesion mechanism. (**i**) Nepenthes-inspired structure design of the hydrogel interface layer. α and β represent the cone angle of microgrooves and the wedge angle of microcolumns, respectively. (**j**) Electrical architecture of the NIH hybrid system. (**k**) Methylene blue droplets on NIH layer (**i**) and undergo directional transport (**ii**). (**l**) System/skin coupling during running (**i**,**ii**) and hydrogel/electrode interface under bending (**iii**). Scale bars: 25 µm (**e**), 4 cm (**f**), 5 mm (**k**,**l**(**iii**)), 50 mm (**l**(**i**)), 10 mm (**l**(**ii**)). Copyright 2024, John Wiley and Sons.

In recent years, the design of electrophysiological sensors has also trended towards higher sensitivity and interference resistance. Researchers have designed nepenthes-inspired microstructures on the surface of dual-network hydrogel electrodes, which significantly improve the signal-to-noise ratio of the signal and reduce the effect of motion artefacts, thereby obtaining high-quality ECG waveforms and heart rate recordings (Figure 4) [74]. These improved high-quality time series signals are commonly used for periodic pattern recognition and anomaly detection in deep learning models, such as real-time monitoring of arrhythmias or analysis of neural signals [46].

### 2.4. Optical Sensors: From Vision to Biometric Information

Optical signals are characteristic information (e.g., refractive index, absorbance, change in light intensity, etc.) generated by the interaction between a biological tissue and light. Conventional silicon-based or glass-based optical sensors cannot meet the requirement of flexibility. Researchers have developed optical sensing interfaces based on waveform optical waveguide materials (e.g., PDMS [76], hydrogels [77]), quantum dots, and nanoparticles [78]. Atomic-scale materials are quantum-limited, which leads to unique optoelectronic properties (e.g., strong optical coupling, multi-exciton generation, tunnelling, etc.). In addition, hybrid perovskite materials have shown excellent optoelectronic properties that have attracted attention [79]. Due to the high degree of freedom in material processing, perovskite is available in a variety of low-dimensional structures, which is promising in flexible electronics. Applications of optical sensors in e-skin include UV monitoring [80], pulse oximetry [81], and photoplethysmography [82] (PPG). Data from these sensors come in the form of time series and frequency-domain signals that can reflect the optical properties of biological tissues or the environment. By analyzing the PPG signals and triaxial accelerometry through deep learning, changes in blood flow velocity or heart rate can be accurately calculated to realize the early detection of atrial fibrillation [81].

More importantly, optical sensors can be integrated with other types of sensors, such as pressure and temperature sensors, to form multimodal sensing networks. This data fusion technique opens up new ideas for comprehensively evaluating complex object properties as well as human physiological states. For example, by integrating optical and mechanical sensing information, researchers have developed an optical artificial skin that is highly sensitive to external mechanical compression and strain. It is more robust and transparent, with a larger sensing area and superior mechanical properties [83].

## 3. Characterization of Electronic Skin Sensing Data

### 3.1. Higher Dimensionality

Data from e-skins are typically stored in high-dimensional arrays. For example, arrays of pressure sensors can provide information on distribution in two dimensions. When combined with multiple sensors, the data dimensions can be extended to three or more dimensions. This multidimensional nature imposes extreme computational demands for data processing. Unlike static image data, the data generated by e-skin not only reflect spatial patterns but also contain dynamic changes, creating unique time-domain features. In high-resolution tactile pressure-detection arrays, data at a single time point can reach hundreds of thousands of dimensions. The data size grows exponentially when data acquisition is performed at kilohertz sampling rates [28].

Traditional linear dimensionality reduction methods, such as principal component analysis (PCA) or linear discriminant analysis (LDA), can reduce the dimensionality of the data but are ineffective at capturing the nonlinear features present in high-dimensional data [84]. Deep learning models, particularly convolutional neural networks and models based on attention mechanisms, show significant advantages in extracting complex spatial pattern features [10,85]. However, the number of parameters in transformer models based on the attention mechanism often exceeds 10^6^. Optimization strategies such as pruning models or binarized networks can be used in the embedded deployment style [86]. Moreover, the presence of high-dimensional data opens up the possibility for multimodal feature extraction. Ye et al. proposed a bimodally coupled multifunctional haptic sensor for non-contact gesture recognition and material identification to address challenges posed by signal interference and high power consumption in multimodal collaboration [87]. The sensor symmetrically integrates capacitive and friction electric sensors, using an energy-complementary approach to reduce energy consumption and effectively prevent signal interference. Using deep learning techniques, the sensor can obtain information about material properties (such as hardness, softness, and deformation) from the detected pressure, perfectly recognizing a wide range of different materials.

However, while high-dimensional data offer possibilities for multimodal feature extraction, they are also constrained by a number of factors. From the perspective of the physical design of the e-skin device, the size and compactness of the sensors limit the spatial resolution of the data acquisition. Transmission bandwidth and storage capacity limit the data transfer and storage capacity (typically <1 GB), low power requirements (typically <10 mW) constrain the deployment of complex algorithms, and thermal effects of the flexible substrate further constrain the performance of the computational units [88].

In terms of application scenarios, different applications have different requirements for real-time data processing and accuracy. In real-time-sensitive scenarios such as medical health monitoring, e-skin requires real-time processing of physiological data to provide timely health warnings. The transmission delay of high-dimensional data may affect the timeliness of anomaly detection, e.g., cardiac ultrasound imaging requires processing speeds of 30 fps or more to meet clinical diagnostic needs. Moreover, in complex real-world environments, e-skins may be subject to multiple interferences, such as electromagnetic interference and ambient temperature variations. To address the above issues, recent research proposes a layered energy management strategy: an event-driven sampling mechanism is used in the signal preprocessing stage, such as frame-based sensor arrays, where only pixels that undergo changes in light intensity can generate data [89]. This design reduces the 16,000 data points collected every 50 microseconds to 200, a 98% reduction in data volume. Introducing a pulsed neural network in the feature extraction session reduces the computation of convolutional operations by 75% using spatio-temporal sparse coding [90]. In addition, a distributed architecture based on federated learning allows for 90% of the model training to be carried out in the cloud, requiring only lightweight inference (<100 kB memory footprint) to be performed locally, significantly reducing end-side power consumption [91].

### 3.2. Temporal Characterization and Dynamic Dependencies

The temporal nature of e-skin data is primarily reflected in the monitoring of bioelectrical signals (e.g., ECG and EEG), motion sensing data, and temperature variations. The dynamic changes in these signals contain information related to health status, tactile patterns, and environmental changes. For instance, certain pathological features in ECG signals (e.g., arrhythmia) may only appear during specific time periods or periodic trends, requiring the analysis of long time series for accurate detection [92,93]. Additionally, activity patterns in EEG signals (e.g., alpha or gamma waves) are closely linked to sleep stages and neural states, and extracting their features requires modeling temporal relationships [94].

Compared to traditional static signals, e-skin time series data exhibit pronounced non-stationarity. This non-smoothness arises from various factors such as sudden temperature changes in the environment, artifacts caused by poor sensor contact, and signal distortion during movement, all of which lead to fluctuations in the time series data. This complexity challenges traditional linear analysis methods (e.g., Fourier transforms), which often struggle to capture the nonlinear dynamic features present in such signals [94,95].

To tackle the challenges of analyzing complex non-stationary time series data, data analysis methods must be robust enough to ensure stable and reliable results. Deep learning techniques, particularly recurrent neural networks (RNNs), long short-term memory networks (LSTMs), and their variants, have shown significant advantages in modeling the dynamic relationships within time series data. These methods have been effectively applied to e-skin data, aiding in the interpretation and understanding of complex waveforms and signal patterns [96,97].

### 3.3. Noise and Artifacts

Electronic skin signals are often influenced by various factors during acquisition, leading to significant noise and artifacts in the data. These interferences can arise from external sources, such as electromagnetic noise, temperature fluctuations, and humidity changes, as well as internal factors like poor contact between the electrodes and the skin. Furthermore, motion-induced changes in sensor positioning can distort the signals. For example, EMG signals frequently contain artifacts caused by electrode slippage or environmental noise, which lower the signal-to-noise ratio and complicate signal processing and feature extraction [72].

To mitigate noise and artifacts in e-skin signals, researchers employ various noise reduction techniques. Traditional methods, such as low-pass filtering and wavelet transforms, can effectively remove high-frequency noise. Wavelet transforms, in particular, are advantageous for preserving essential signal features when working with non-stationary signals; however, their adaptability in complex noise environments is limited. In recent years, deep learning techniques have introduced innovative methods for noise reduction. Self-supervised learning has also been applied to pre-training tasks to construct latent feature distributions of unlabeled data, enhancing signals and suppressing artifacts [98,99]. Variational Autoencoders (VAEs) have also been employed to reconstruct clean signals by learning latent feature distributions [100].

### 3.4. Multimodal Characteristics and Fusion Challenges

A significant advantage of e-skin is its multimodal sensing capability, which enables the simultaneous collection of various types of information, such as pressure, temperature, bioelectrical signals, and chemical data. This multimodal sensing not only provides individual physical data but also reveals more complex patterns through their interactions. For instance, in medical applications, the combined analysis of bio-signals such as pressure, temperature, and body fluids enables more accurate assessments of a patient’s inflammatory state or circulatory abnormalities [101]. Additionally, in smart prosthetics, the integration of bioelectrical signals with haptic feedback creates a more natural and intuitive human–machine interaction experience [102]. However, differences in the dynamic range, sampling frequency, and noise characteristics of different signals pose challenges for efficient multimodal data fusion.

The fusion of multimodal data involves addressing several key challenges. First, data between different sensors will often have different timestamps and sampling rates. The data often suffer from time synchronization, leading to timing alignment difficulties. For example, pressure sensors may be sampled at hundreds of times per second, while temperature sensors may have much lower sampling frequencies. To achieve accurate data alignment, complex interpolation and synchronization algorithms (e.g., Dynamic Time Warping (DTW)) may be introduced [103]. These algorithms require more computational resources and computational cycles, leading to increased energy consumption. This not only affects the accuracy of the data but also increases the difficulty of data processing. Second, differences in data information represented across modalities increase the complexity of feature fusion. Pressure signals are typically stored as two-dimensional matrices, while bioelectrical and chemical signals are represented as one-dimensional time series. This disparity complicates the uniform modeling of features across modalities, potentially impacting the quality of fusion. Moreover, noise in multimodal signals presents significant challenges. Noise in one modality may propagate through the fusion process and distort other signals, reducing the reliability of the final analysis. Most importantly, the interactive nature of multimodal data complicates signal decoupling. In complex sensor circuits or multimodal bioelectronic skins that integrate strain, temperature, and pressure detection, effective decoupling strategies are essential. These strategies must distinguish the independent contributions of each modality while preserving the intermodal interaction characteristics [101,104].

## 4. Deep Learning Methods in Data Analytics

### 4.1. Basic Concepts of Modeling and Different Application Scenarios

E-skin sensing data are characterized by high-dimensionality, temporality, and multimodality. Deep learning techniques have become essential tools for analyzing and interpreting these complex data. Below are several commonly used deep learning technologies and their specific applications in e-skin data analysis. Table 1 provides an overview of how different deep learning technologies can be applied to e-skin data, highlighting their advantages, disadvantages, and applications in wearable e-skins.

#### 4.1.1. Convolutional Neural Networks

Convolutional neural networks (CNNs) are feed-forward neural networks designed to extract features from data with a convolutional structure, such as images and matrices. They perform well in image processing and spatial pattern recognition. Unlike traditional feature extraction methods [114], CNNs are inspired by the process of visual perception [115], and their architecture allows for automatic feature extraction from input data. This eliminates the need for complex manual feature engineering. CNNs consist of the following key components:

- **Convolutional Layer**: This is the core component of a CNN, which is responsible for extracting local features from the input data using multiple filters. Each convolutional kernel slides across the input data to generate a feature map, capturing important spatial information such as edges, corners, and textures [116,117,118]. Due to the local connectivity property, CNNs can effectively reduce the number of parameters and computational complexity.
- **Activation Function**: After the convolutional layer, a nonlinear activation function (e.g., ReLU, Sigmoid, or Tanh) is typically applied to introduce nonlinearity. This enables the network to learn more complex feature representations, capture higher-order patterns in the input data, and improve the model’s expressive power [119].
- **Pooling Layer**: The pooling layer reduces the size and computational complexity of the feature map by downsampling while enhancing the invariance of the features. This helps prevent overfitting and improves the model’s ability to tolerate input deformations such as rotations or displacements.
- **Fully Connected Layers**: After multiple convolutional and pooling layers, the features are flattened and passed through one or more fully connected layers. These layers map the extracted features to the final output, such as classification labels or regression values, allowing the model to make the final decision.

In electronic skin applications, CNNs effectively extract sensor data features (such as pressure and temperature) through hierarchical feature learning, enabling tasks such as surface identification, feature extraction, and health detection [120,121,122]. This makes CNNs highly accurate in analyzing pressure sensor arrays. However, while CNNs excel at handling local features, they may struggle to capture long-range dependencies. Additionally, CNNs require large datasets, and their performance can be affected by the quality and quantity of the input data. As a result, CNNs may not perform well in environments with small datasets.

#### 4.1.2. Recurrent Neural Networks and Their Variants

Recurrent neural networks (RNNs) are a class of neural networks that take sequence data as inputs and recursively process the data in the sequence’s temporal direction, with all nodes connected in a chained fashion. The core feature of RNNs is their ability to dynamically maintain the contextual information of the input sequence through temporal recursive connections. This characteristic enables RNNs to effectively capture temporal dependencies, making them suitable for tasks such as speech recognition and time series prediction [17,123,124]. The basic components of an RNN include:

- **Input Layer**: Receives time series data as inputs, typically shaped as the number of samples, time steps, and number of features.
- **Hidden Layer**: Recursively memorizes and updates information from previous time steps. The hidden state at each time step depends not only on the current input but also on the hidden state from the previous time step. This structure allows the RNN to capture temporal relationships in sequential data.
- **Output Layer**: Generates the prediction result for the current time step based on the hidden state, ensuring continuous information flow.

In gradient computation within RNNs, issues such as gradient decay or explosion may arise, especially when the number of time steps is large or small. To address these challenges and improve RNN performance with long sequential data, long short-term memory (LSTM) networks and gated recurrent units (GRUs) were introduced:

- **Long Short-Term Memory**: LSTM networks introduce memories that share the same shape as hidden states and are used to store additional information [108]. LSTM controls the flow of information via forget gates, input gates, and output gates. The forget gate decides which information should be discarded, the input gate selects the new information to be added, and the output gate controls the output of the hidden state. The LSTM architecture allows it to efficiently capture long-term dependencies in time series data, which is particularly important for physiological signal monitoring and anomaly detection.
- **Gated Recurrent Unit**: A GRU is a simplified version of an LSTM, merging input and forget gates to reduce the model’s complexity [109]. Due to their reduced number of parameters, GRUs are widely used in scenarios requiring faster computational speeds, such as real-time motion pattern recognition.

In e-skin applications, both LSTM networks and GRUs are capable of monitoring physiological signal changes and analyzing health status in real time. The introduction of these models resolves the challenges faced by traditional RNNs in analyzing long sequences, improving both the accuracy and efficiency of the models.

#### 4.1.3. Transformer

A transformer is a neural network architecture based on a self-attention mechanism, which has achieved success in fields such as natural language processing by addressing the long sequence dependency problem through parallel computation and multilayer feature extraction [110]. The key components of the transformer include:

- **Multi-head Self-attention Mechanism**: This mechanism allows the model to compute the relevance of each position in the input sequence, adaptively focusing on information from different positions. This helps the model capture long-range dependencies and context, making it especially well-suited for processing long sequences of data.
- **Feed-forward Neural Network**: After each self-attention layer, there is a feed-forward neural network responsible for enhancing and transforming the representation of each position. This network typically consists of two linear transformation layers and an activation function, allowing the extracted features to have rich expressive power.
- **Layer Normalization and Residual Connections**: Between the self-attention layers and the feed-forward networks, layer normalization is applied to improve training stability and convergence speed, while residual connections help reduce the difficulty of training deep networks.

The encoder–decoder structure of the transformer can handle various types of inputs and outputs, making it suitable for tasks like health monitoring, material analysis, and facial expression recognition [111,125]. In the context of e-skin applications, transformers can effectively integrate data from different sensors, helping to infer complex patterns and improve the handling of multimodal data. However, the complex architecture of transformers requires significant computational resources and memory, and they may not perform as well on smaller datasets compared to other models.

#### 4.1.4. Self-Supervised Learning and Transfer Learning

Self-supervised learning and transfer learning are important strategies for applying deep learning in e-skin technologies. Self-supervised learning allows models to be effectively trained without large amounts of labeled data by designing pre-training tasks. For example, the model can learn by reconstructing or predicting data in a time series, which is particularly useful when data are scarce [112].

Transfer learning, on the other hand, leverages deep learning models that have been trained on large datasets, using the knowledge gained to significantly accelerate the training process for small-sample learning tasks. By providing the model with pre-trained parameters, it can converge more quickly on new e-skin datasets, enabling e-skin technologies to rapidly adapt to different environments and tasks, resulting in improved learning efficiency and accuracy [113].

However, the effectiveness of these methods typically depends on the similarity between the source and target tasks. If the features from the source task cannot be directly transferred to the new task, it may cause a decline in model performance.

### 4.2. Data Preprocessing and Feature Extraction

In deep learning applications for e-skin, data preprocessing and feature extraction are crucial steps to ensure optimal model performance and accuracy. As mentioned earlier, the data generated by e-skin often suffer from issues such as noise, instability, and high dimensionality. Therefore, effective preprocessing must be carried out before feeding the data into the deep learning model to enhance the quality and reliability of the analysis.

#### 4.2.1. Data Cleaning and Noise Reduction

Electronic skin signals are often affected by noise and artifacts, and data cleaning and noise reduction are essential solutions. The sources of noise include electromagnetic interference, poor sensor contact, and environmental factors. To improve signal quality and ensure analytical accuracy, effective noise reduction methods must be applied. Traditional noise reduction methods utilize signal processing techniques to remove noise while preserving the relevant components.

Common noise reduction techniques include low-pass filtering and wavelet transforms. Low-pass filtering effectively removes high-frequency noise while retaining low-frequency components, improving the signal-to-noise ratio. For data from multimodal electronic skin sensors, low-pass filtering helps reduce high-frequency interference caused by poor contact or sensor noise, resulting in more stable signals [126]. Wavelet transforms decompose signals and remove noise through time–frequency analysis, making them suitable for processing non-stationary signals and those with abrupt changes or localized features. These techniques are commonly used to remove artifacts caused by environmental factors or motion and are particularly effective for sensor data with large fluctuations, such as strain and skin temperature signals [127].

While traditional noise reduction methods are effective, deep-learning-based techniques offer greater potential with the advancement of deep learning technologies. Self-supervised learning, convolutional neural networks, and generative adversarial networks can automatically learn the spatial and temporal characteristics of noise and effectively remove it, especially excelling in high-dimensional and multimodal data processing [128,129,130]. However, despite the higher accuracy of deep learning methods, traditional approaches remain effective and widely used for noise reduction, particularly when real-time processing and computational resources are limited.

#### 4.2.2. Feature Extraction

Feature extraction is a crucial part of data preprocessing, as it transforms raw signals into meaningful features that can be utilized by deep learning models. Effective feature extraction helps the model extract key information, enhancing learning capability and improving prediction accuracy.

Electronic skin signals are typically temporal and multimodal, requiring feature extraction methods that reflect their multidimensional characteristics. In time-domain analysis, commonly used statistical features such as the mean, standard deviation, skewness, and kurtosis provide preliminary input data for deep learning models [28,131]. These features reveal the fluctuation patterns of the signal and assist in monitoring changes in various physiological states. Additionally, if multiple time-domain features of uncertain validity need to be extracted before initial training, principal component analysis (PCA) is often effective in selecting the correct features [132].

For periodic signals, frequency-domain feature extraction becomes essential. In high-frequency signals such as sound and texture, time-domain features may not fully describe the signal. By applying the Fourier transform, time-domain signals can be converted into the frequency domain, revealing the signal’s frequency components [133,134]. Frequency-domain analysis allows for differentiation between low- and high-frequency components, providing more detailed feature inputs for deep learning models. Furthermore, many electronic skin signals exhibit variations in both the time and frequency domains, so traditional time-domain or frequency-domain methods may fail to capture the full dynamic characteristics of the signals. To address this, time–frequency analysis methods such as the short-time Fourier transform (STFT), wavelet transform (WT), and Wigner–Ville distribution (WVD) can be used to extract features from both the time and frequency dimensions [135,136,137].

## 5. Key Applications of Deep-Learning-Powered E-Skin

As an innovative technology that mimics and even surpasses the perceptual capabilities of human skin, e-skin can collect multidimensional physiological and environmental data in real time. Thanks to the pattern recognition and feature learning capabilities of deep learning, e-skin plays a critical role in processing these complex data and enabling intelligent decision-making. Table 2 shows representative e-skins applying deep learning techniques in recent years.

### 5.1. Cardiovascular Disease Monitoring

Active and continuous monitoring of blood pressure (BP) is one of the most fundamental practices in modern medicine, playing a crucial role in preventing deaths related to cardiovascular diseases [158]. Currently, physicians rely on traditional cuff sphygmomanometers to measure static values of systolic blood pressure (SBP), diastolic blood pressure (DBP), and mean arterial pressure (MAP) [159]. However, these devices do not allow for the continuous and uninterrupted monitoring of a patient’s hemodynamics in daily, ambulatory, and nocturnal settings [160].

Existing cuffless blood pressure monitoring methods, which use acoustic, pressure, or optical modalities, face challenges in simultaneously achieving accurate blood pressure signal capture while maintaining good skin compatibility [161]. To address these issues, researchers have designed a self-adhesive, low-impedance graphene electronic tattoo (GET) that adheres firmly to the skin, ensuring stable positioning over time [162]. This device enables stable sensing of hemodynamic parameters directly from the arteries by measuring bioimpedance (Bio-Z), which remains relatively stable. Using a deep learning approach, the obtained Bio-Z data are modeled to create a reliable BP estimation model. The system operates with high fidelity, causing no disturbance to the patient, and provides accuracy comparable to that of a Class A wearable blood pressure measurement device, offering a promising solution for continuous wearable blood pressure monitoring.

### 5.2. Elderly Care

**Figure 5:**
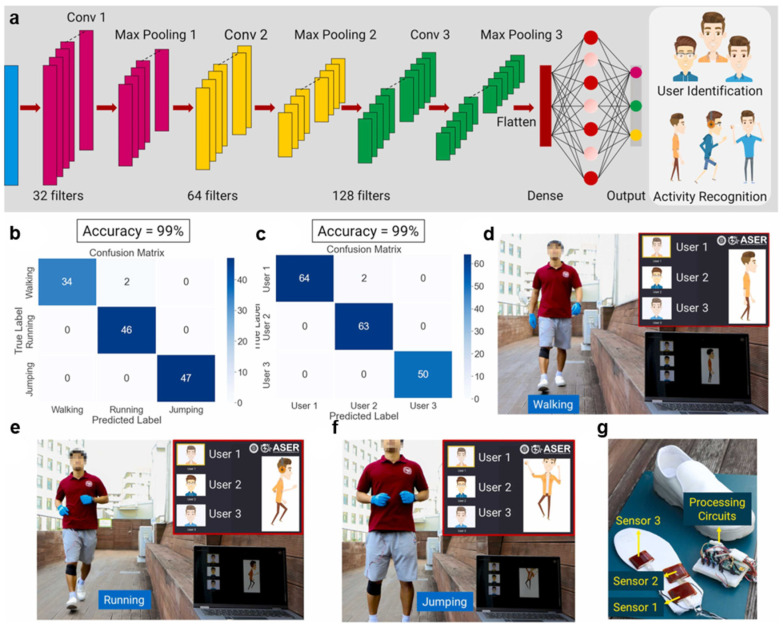
Human activity recognition and user identification using the deep learning method [34]. (**a**) A 1D-CNN system architecture for activity recognition and user identification. Confusion matrices for (**b**) activity prediction (99% accuracy) and (**c**) user prediction (99% accuracy). Photographs of user 1 during (**d**) walking, (**e**) running, and (**f**) jumping, with insets showing correct identification and activity. (**g**) Photograph of the processing circuit and TENG sensors on the shoe insole for data collection. Copyright 2023, Elsevier.

E-skin can also monitor the activity level, posture, and sleep quality of older adults to help provide safe care. Park et al. developed a double-layer nanofibrous electric nanogenerator (TENG) using an electrostatic spinning process, placed it on an insole, and analyzed its friction electric sensing data through deep learning techniques (Figure 5) [34]. This device recognizes the user’s activity status through the compression of the insole and can respond promptly if an elderly person falls or exhibits abnormal activity. In addition, Xu et al. developed an e-skin based on metallic fabrics and natural friction electric technology for detecting electrical signals generated by friction between the shoe sole and the ground or between the skin and fabric when the body is in motion or undergoing changes in its physical state [163]. People often make movements such as rolling over, bending their legs, or raising their hands while sleeping, and these movements typically occur not individually but as a combination of several actions. When these movements happen, friction occurs between the hands and clothing and the legs and the bed, generating electrical signals that change in sequence over time. These data are analyzed by a one-dimensional convolutional neural network, which can effectively track sleep states. It can remotely recognize the body’s state and alert caregivers to changes in physiological signals due to unexpected events.

### 5.3. Sweat Monitoring

Sweating is the body’s physiological response to the movement of fluids from sweat glands to the skin’s surface. Individual sweat levels can serve as an important physiological marker of the body’s health, comfort, emotional state, and exertion. The current wearable sweat monitoring process can be divided into two parts: sweat collection and analysis. Sweat collection primarily relies on microfluidic devices. Microfluidic sensors use multilayered structures to collect sweat, which implies a complex manufacturing process, high production costs, and the use of rigid materials [164].

Colorimetric analysis is an optical method that uses a specific ingredient to react with a reagent under certain conditions to produce a colored compound. Light is then passed through the colored solution and the sample to be measured. The shade of the colored solution is compared to that of a standard solution. However, the safety of this method requires further consideration [165].

**Figure 6:**
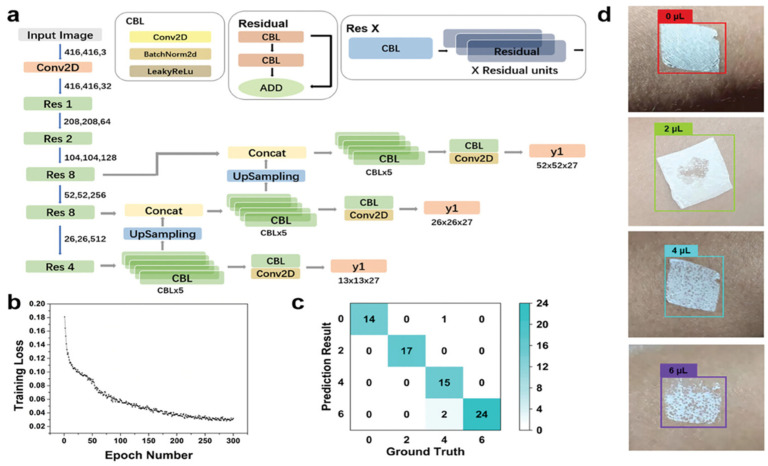
Facial EMG monitoring by PLPG and machine learning for emotion analysis [166]. (**a**) Schematic diagram of the YOLOv3 algorithm backbone network consisting of three upsamples that output three feature maps: y1, y2, y3. (**b**) YOLOv3 training loss vs. epochs. (**c**) Confusion matrix for 4 perspiration categories. (**d**) Images of perspiration categorization results. Copyright 2023, John Wiley and Sons.

To address these challenges, Chen et al. developed a colorimetric electronic skin for smart sweat monitoring [166]. This e-skin consists of a polyurethane nanoweb and an object detection algorithm, YOLOv3. The polyurethane nanoweb has a 44% porosity and capillarity, making it a low-cost colorimetric indicator. When sweat is absorbed, the nanoweb’s volume expands by 362.37%, and its light transmission changes by 277.78%. The researchers used a capillary-action-based finite-element model to explain the change in light transmission, created a database of 735 images, and analyzed the sweat data using the YOLOv3 algorithm. The test results successfully recognized the sweat volume with 97% accuracy (Figure 6). This research offers a new direction for healthcare applications.

### 5.4. Texture Recognition

**Figure 7:**
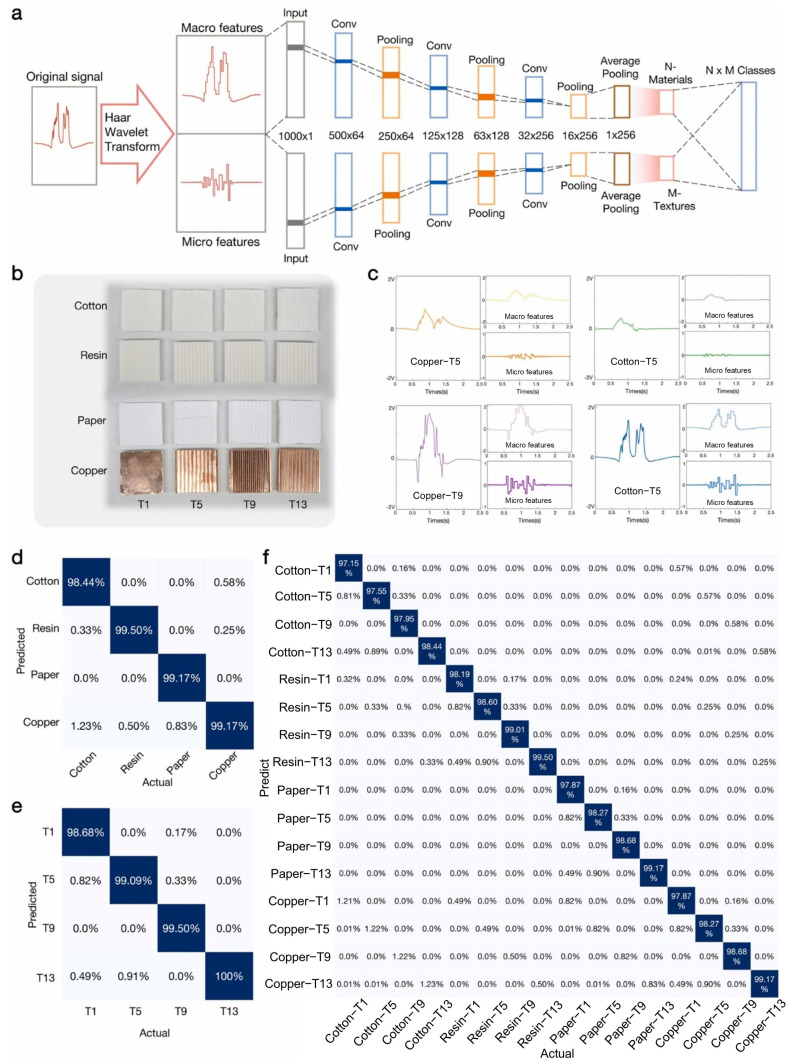
Signal decoupling and simultaneous recognition model [45]. (**a**) Architecture of the decoupling and 1D-CNN-based recognition model for feature extraction and classification. (**b**) Sixteen standard objects from the cross-pairing of four materials (copper, cotton, resin, paper) and four textures. (**c**) Sample sensing signals and corresponding decoupled features. (**d**) Confusion matrix for material recognition (4 materials). (**e**) Confusion matrix for texture recognition (4 textures). (**f**) Confusion matrix for merged recognition of the 16 objects in (**b**). Copyright 2022, Elsevier.

The haptics of electronic skin enable intelligent robotic systems and prosthetics to perform precise motion trajectory planning and interact naturally with humans and the environment. Traditional unimodal tactile sensors can only detect mechanical stimuli and lack the ability to sense material properties, while multimodal sensors face challenges in decoupling their signals, which may interfere with each other [167,168]. Song et al. developed a novel tactile sensor using friction nanogenerators that achieves multimodal sensing with a single mechanism [45]. Thanks to its inherent contact electrochemical properties and the grating structure used, the macroscopic features (such as amplitude, trend, and envelope) of the output signal can be utilized for material identification, while the microscopic features (such as frequency, change points, and variance) can be used for surface texture recognition. The haptic integrated system based on this sensor enables real-time and synchronized recognition of materials and textures. The sensor’s touch is converted into electrical signals, which are then decoupled into macro-features and micro-features through signal processing. These features are fed into a convolutional neural network for pattern decoding and feature extraction. The model can accurately predict materials and textures with 99.07% and 99.32% accuracy, respectively (Figure 7). Additionally, the system can recognize special raised points of Braille, identify Braille information, and distinguish the material of Braille. This capability can significantly help blind and visually impaired individuals better understand Braille on various infrastructures.

### 5.5. Gesture Recognition

**Figure 8:**
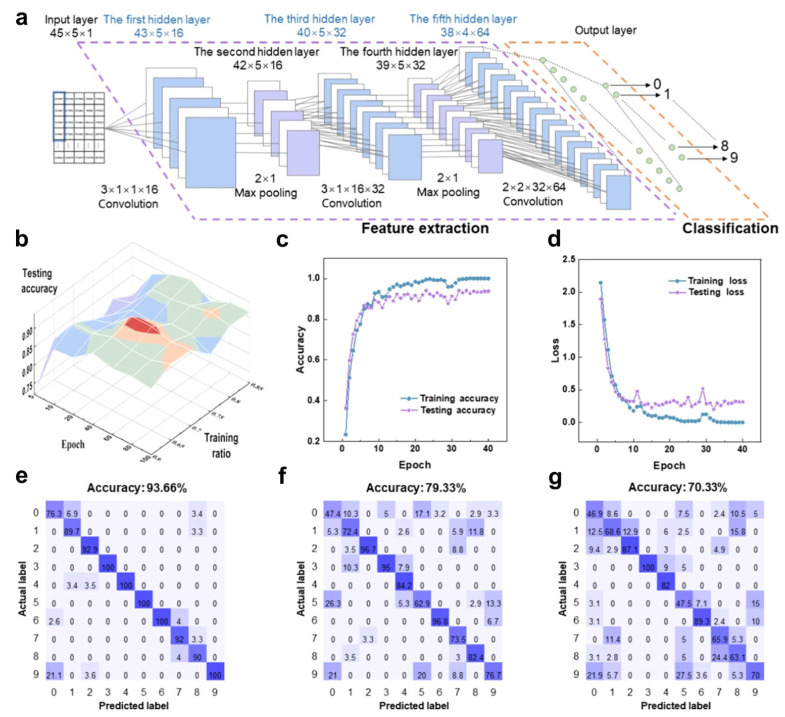
Realization of hand gesture recognition by deep-learning-based algorithm [10]. (**a**) Process of hand gesture recognition with deep convolutional neural networks (DCNNs). (**b**) Three-dimensional plot of test accuracy vs. epochs and training ratios. (**c**) Accuracy rate transition with increasing epochs. (**d**) Loss rate transition with increasing epochs. (**e**) Confusion matrix for DCNNs. (**f**) Confusion matrix for support vector machines. (**g**) Confusion matrix for K-nearest neighbors. Copyright 2024, John Wiley and Sons.

Gesture interaction, as an important natural interface, allows for communication with machines through instinctive and perceptual behaviors. It is one of the areas in human–machine interaction that requires further breakthroughs [169]. Various techniques, including machine vision, infrared, radar, ultrasound, and electrical signals, have been widely studied and explored for gesture recognition. By introducing a microscale incompatible phase into a polymer network, Sun et al. developed a highly elastic and durable ionogel sensor [10]. This sensor is characterized by a high sensitivity, broad response range, and high durability, making it ideal for accurately monitoring human activity. Using this strain sensor, gestures corresponding to the numbers 0 to 9 were precisely captured from five participants with different hand characteristics, creating a reliable dataset. The gesture sensor signals are compensated using a deep convolutional neural network to form a system capable of dynamic gesture recognition (Figure 8). A system based on this model can analyze both the temporal and spatial characteristics of the sensor data, helping to better understand the dynamic process of gestures.

### 5.6. Emotion Recognition

**Figure 9:**
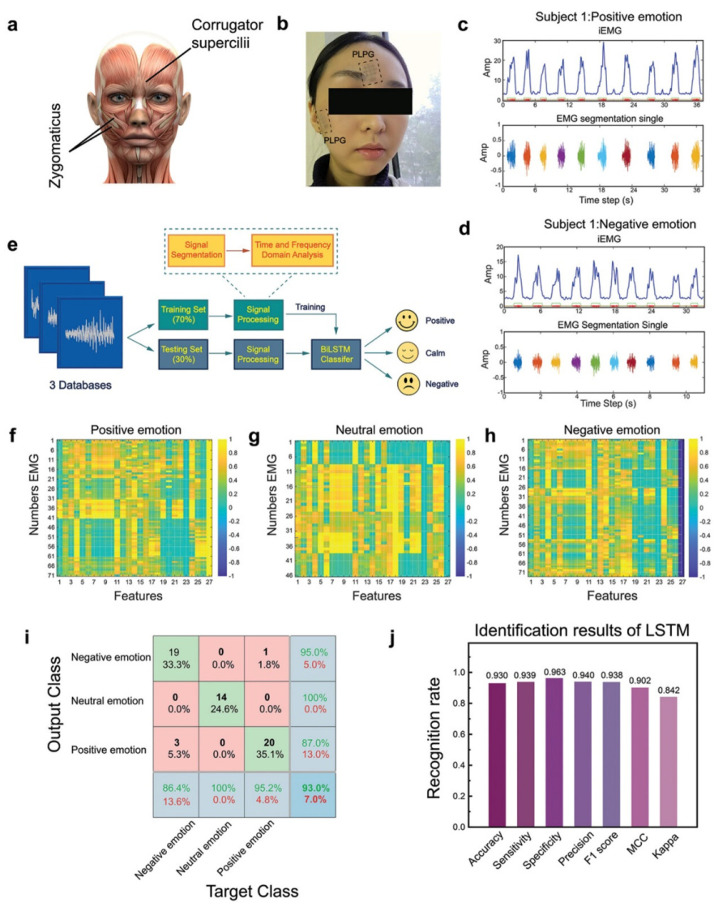
Facial EMG monitoring by PLPG and machine learning for emotion analysis [11]. (**a**) Main muscles for emotion expression. (**b**) PLPG with M-3 pattern electrodes for fEMG acquisition. (**c**,**d**) Representative fEMG signals and extracted integrated EMG for positivee (**c**) and negative (**d**) emotions. (**e**) Machine learning flowchart for emotion classification. (**f**–**h**) Thermogram of fEMG correlation coefficients for positive (**f**), neutral (**g**), and negative (**h**) emotions, with classification labels in the 27th column. (**i**) Confusion matrix for classification accuracy. (**j**) LSTM identification results. Copyright 2024, John Wiley and Sons.

Emotions are important indicators of people’s internal states, and understanding emotions can help predict human behavior and provide appropriate feedback. Currently, speech signal analysis, image-based facial action coding systems, and electrophysiological signal analysis are commonly used for emotion recognition [170,171,172]. Among these methods, electrophysiological signals offer high accuracy and sensitivity. Du et al. reported a 40 nm thick film based on a photolithographic double-network conductive polymer mediated by a graphene layer [11]. When applied to the zygomatic bone, this film can monitor subtle facial movements induced by visual stimuli and capture corresponding facial electromyographic signals (fEMG). After preprocessing the fEMG signals using a moving average filter, they were classified into three emotional categories: positive, negative, and neutral. A bidirectional long short-term memory network was used to build the classification model, achieving an accuracy of 93%. Through accurate data acquisition and machine learning, the model effectively identified and analyzed the emotional experience’s potency (positive or negative) and the intensity of the emotion (Figure 9).

### 5.7. Virtual Shopping

**Figure 10:**
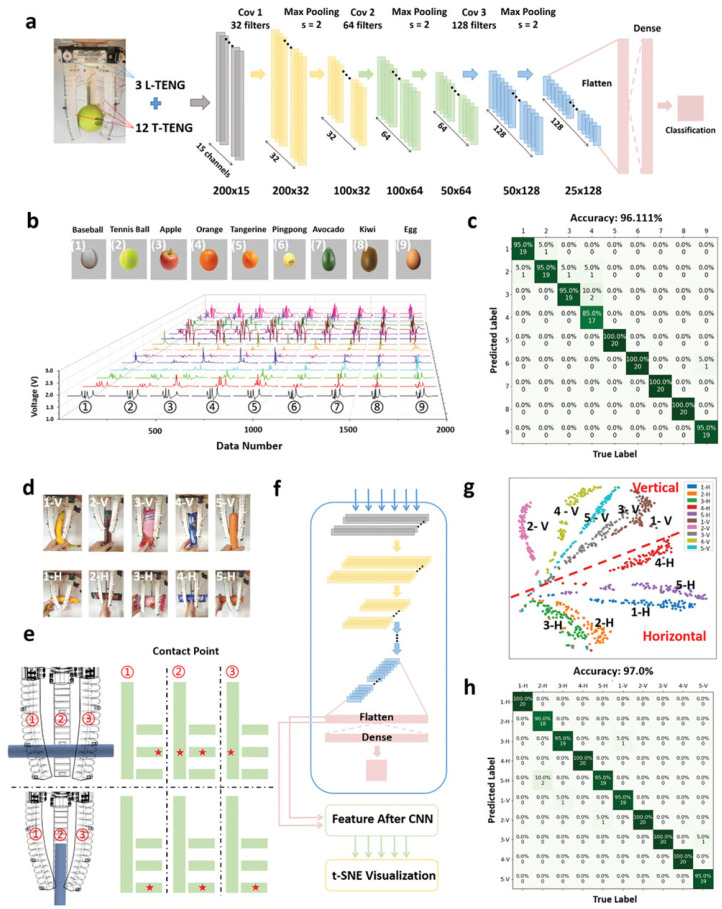
ML-enabled automatic grasped objects recognition system [173]. (**a**) A 1D-CNN framework. (**b**) Fifteen-channel spectra from TENG system for 6 spherical and 3 oval objects. (**c**) Confusion map for spherical and oval objects. (**d**) Manipulator grasping 5 elongated objects vertically and horizontally. (**e**) Deformation and contact map of manipulator with T-TENG patches. The marks of five-pointed star represent the contact positions on the T-TENG sensor patches integrated on three pneumatic fingers. (**f**) t-SNE visualization framework. (**g**) t-SNE results for vertical and horizontal grasps. (**h**) Confusion map for 5 elongated objects at two grasping angles. Copyright 2023, John Wiley and Sons.

With the rapid development of the Internet and logistics, online shopping has become an indispensable part of our daily lives, offering great convenience and enabling us to collect desired products without leaving home. To better enhance the customer experience and provide an immersive shopping experience in a virtual store using VR technology, Gong et al. reported an intelligent soft robotic arm supported by tactile/length friction nanogenerator (T-TENG, L-TENG) sensors and polyvinylidene difluoride (PVDF) pyroelectric temperature sensors [173]. The pyroelectric sensors allow the robotic arm to capture the temperature distribution of an item. Additionally, the robotic arm, equipped with T-TENG and L-TENG sensors, has 15 channels for data collection. When an item is grasped, the stimulus applied to the inner surface of each finger generates three-dimensional sensory information, including the contact position and area of the three contact surfaces. The deformation of each finger also provides information about the size and shape of the object, especially for asymmetrical objects. Through deep learning, hidden information such as the contact position, contact area, and bending angle is computed from the sensing signals, enabling the automatic recognition of 28 different object shapes with an accuracy of 97.143% (Figure 10). By utilizing IoT and AI analytics, a virtual store based on digital twins was successfully implemented, providing users with real-time feedback on product details.

## 6. Challenges and Prospects

In recent years, e-skin technology has made rapid progress in the fields of health monitoring, intelligent robotics, and human–computer interaction, yet its further development still faces multidimensional technical challenges. This chapter systematically analyzes the current core bottlenecks and proposes domain-specific solutions and development routes (Table 3).

One of the core issues facing e-skin technology is the challenge of data standardization and model generalizability. Different devices and sensors differ significantly in terms of hardware architecture, sampling frequency (0.1–10 kHz), signal resolution (8–16 bit), and data format (CSV/JSON/Binary) [174]. It has been shown that when sensors with different data protocols, e.g., IEEE 802.15.4 [175] vs. OSI model, are trained cross-platform, the accuracy of the ResNet-50 model in a tactile recognition task decreases by 20% [176]. This fragmentation severely limits cross-platform data sharing and interoperability and hinders the creation of large-scale multimodal datasets. In addition, the lack of standardization directly affects the efficiency of deep learning models. E-skin data are usually high-dimensional, multimodal, and non-stationary, which makes it difficult to generalize existing models effectively. In a pressure sensor data standardization experiment, training a convolutional neural network using datasets from different vendors resulted in a cross-validation rate that varied as much as 23% [177]. This discretization stems from the lack of uniform data preprocessing specifications, such as baseline correction methods and noise filtering thresholds. This problem is more prominent in open environments, where sensor data may be disturbed by environmental noise or changes in user behavior, thus reducing the adaptability of existing models. To solve such problems, standardized protocols covering the whole chain of sensor calibration, feature extraction, and data transmission need to be established. A dynamic adaptation framework based on meta-learning can effectively reduce the generalization error of classification models on uncalibrated devices by embedding domain-adaptive modules in the model architecture [176]. Meanwhile, the development of a smart sensor interface protocol compliant with the IEEE 21451-2 standard [178] will also effectively improve the efficiency of multi-source data fusion.

Another major bottleneck limiting AI e-skins is the lack of high-quality labeled datasets. Multimodal sensors enable e-skin systems to collect large amounts of physiological and environmental data, but traditional supervised learning faces exponentially increasing labeling costs that greatly limit the size of available datasets. As a result, lab-based self-constructed datasets tend to be small samples. Their accuracy in terms of surface EMG signals, for example, is up to 5–7 min of expert annotation time for a single action recognition sample, and existing open datasets such as Ninapro DB5 contain only 53 types of action data from 10 subjects [179]. The lack of data leads to insufficient model training, negatively impacting its performance and practical large-scale applications [176]. The emerging solution combines unsupervised representation learning with weakly supervised fine-tuning techniques: contrast learning is first used to extract generic features from unlabeled signals of order 10^6^, and then task-specific migration is achieved with a small amount of labeled data (<10%). This approach achieved 87.2% accuracy in a continuous gesture recognition task, a 19% improvement over purely supervised learning [180].

Higher computational performance is also required for tasks with larger model parameters. In addition, real-time processing and privacy are unavoidable topics. Emerging technologies such as edge AI and cloud AI technologies can be further developed. They can be integrated into e-skin systems to solve these problems. Edge AI refers to the deployment of deep learning models and algorithms directly on the e-skin. This approach allows for real-time processing and decision-making on the device and can effectively reduce the need for data transmission bandwidth and the latency incurred by communicating with the cloud. In addition, the non-circulation of data can maximize the protection of user privacy. Cloud artificial intelligence technology, on the other hand, utilizes the more powerful computational and storage capabilities of remote servers to analyze data from e-skins. By incorporating a distributed learning framework for federated learning and privacy-preserving techniques, it is possible to share a larger dataset while ensuring privacy security [181].

The interpretability of AI models has also emerged as a pressing issue. While many deep learning models in medical and health monitoring applications demonstrate impressive predictive accuracy, their “black box” nature lacks intuitive explanations for their decision-making processes. This lack of transparency makes it difficult for users, particularly doctors and patients, to fully trust these technologies [182]. To address this, future research should emphasize the development of more transparent AI methods, such as integrating attention mechanisms, interpretable neural networks, or causal inference models, to make the decision-making process comprehensible [183,184]. Studies have shown that combining physically inspired attention mechanisms with deep learning can significantly improve interpretability [185]. For example, in a diabetic foot ulcer prediction model, the introduction of a spatio-temporal attention module based on the propagation law of biological impedance enables the localization of key pathological features with millimeter-level accuracy, while providing decisions that are consistent with clinical experience [186].

Lastly, a relatively underexplored area is the enhancement of AI’s behavioral prediction capabilities. Currently, most AI applications are focused on classifying or analyzing collected data, lacking the ability to predict users’ future behavior [187]. For example, if a prosthetic device equipped with e-skin can predict the user’s next movement based on historical behavioral patterns, it could preemptively adjust and prepare the prosthetic for the action. This capability has broad potential in applications like posture prediction, motion assistance, and rehabilitation training, further elevating the intelligence of e-skin systems in human–computer interaction. Introducing neuromorphic computing into the e-skin system can break through this bottleneck; using a spiking neural network to process millisecond tactile timing signals, an ultra-low-latency response of 93 ms was achieved in a prosthetic grip prediction task, improving the real-time performance by a factor of 5 compared with the traditional LSTM model [188].

**Table 3:** A summary of the challenges and prospects of e-skin.

Category	Challenges	Solutions and Prospects	Reference(s)
Data Standardization and Model Generalizability	Differences in architecture, sampling frequency, and data formats Decreased model accuracy in cross-platform training High-dimensional, multimodal, non-stationary data	Establish standardized protocols for sensor calibration, feature extraction, and data transmission Implement dynamic adaptation frameworks based on meta-learning Promoting the IEEE Smart Sensor Interface Protocol Development of dynamic optimization algorithms for multi-source data fusion	[174,176,177]
Lack of High-Quality Labeled Datasets	High labeling costs for large datasets Small sample sizes limit model training	Combining unsupervised learning and weakly supervised fine-tuning techniques Combining contrast learning with small labeled datasets for better performance Improve methods for sharing datasets	[176,179,180]
Computational Performance	Larger model parameters require high computational resources Need for real-time processing and privacy	Implement edge AI for real-time processing and decision-making on e-skin Leveraging cloud AI for big dataset analysis with federated learning and privacy-preserving technologies Further development of edge AI for real-time performance Protecting privacy through distributed learning and non-circulating data	[181]
AI Model Interpretability	Lack of an intuitive explanation of the decision-making process	Developing transparent AI models with attention mechanisms, interpretable neural networks, or causal inference models Focusing on AI transparency to build trust in medical and health applications Integrating biologically inspired attention mechanisms	[182,183,184,185,186]
Behavioral Prediction	Lack of ability to predict future user behavior, limiting AI capabilities in dynamic scenarios	Introducing neuromorphic computing to predict user actions based on historical data, using spiking neural networks for low-latency responses Enhancing prediction capabilities for prosthetics, posture prediction, and rehabilitation	[187,188]

## Figures and Tables

**Table 1:** Comparison of different deep learning technologies.

Technology	Advantages	Disadvantages	Applications	Reference(s)
CNNs	Automatic extraction of spatial features	Difficult to capture long-range dependencies	Demonstrates high classification accuracy for the analysis of pressure sensor arrays	[105,106,107]
	Parameter sharing reduces computation	Requires large amounts of labelled data	Enables tasks such as surface identification, feature acquisition, and health detection	
	Suitable for image/matrix data			
RNNs/LSTM/GRU	Highly capable of temporal modeling	Gradient vanishing/exploding (RNNs)	Real-time monitoring of changes in physiological signals to analyze health status	[108,109]
	Dynamically maintains contextual information Suitable for sequential data	Slow training	Solves the dilemma of traditional RNNs in long sequence analysis and improves accuracy and efficiency	
		Difficult to parallelize		
Transformer	Parallel computation is efficient	High consumption of computational resources	Integrates data from different sensors to infer complex patterns	[110,111]
	Self-attention captures long-range dependence	Easy overfitting for small data	Improves the processing of multimodal data	
	Strong multimodal fusion			
Self-supervised Learning	No need for large amounts of labelled data	Pre-training tasks are designed to be sensitive	Enables models to be trained efficiently by designing pre-training tasks when there are insufficient data	[112]
	Generic features learned through pre-training tasks	Migration effects are dependent on task relevance	Suitable for small-sample learning tasks to speed up the training process	
Transfer Learning	Reduce data requirements	Dependent on similarity between source and target tasks	Cross-scene health monitoring model migration	[113]
	Reuse pre-trained model knowledge	Possible negative migration	Sensor disparity adaptation	

**Table 2:** Representative studies that used DL-powered electronic skin for tasks.

Category	Targeted Parameters	Number of Model Parameters	Number of Sensor Channels	DL Models	Learning Objectives	Year	Reference
DL for data processing	Humidity, temperature, pressure, and UV	4	10^5^	LSTM	Multi-signal decoupling	2022	[138]
	Pressure, temperature	2	10^5^	CNN	Temperature and pressure mapping Signal decoupling	2024	[64]
	Ionic liquids, photovoltaics, conductive fabric signals	3	10^5^	Transformer	Multi-signal decoupling	2024	[125]
DL for healthcare	Directional flow of air and air vibration during respiratory activity	1	10^6^	CNN	Cough diagnosis	2022	[122]
	Biomarkers of wound exudates	5	10^6^	CNN	Wound healing monitoring	2023	[121]
	EEG	2	10^6^	LSTM	Epileptic seizure detection	2023	[124]
	Friction from motion	3	10^6^	CNN	Motion status monitoring	2023	[34]
	Thiram residues	1	10^4^	CNN	Food safety testing	2025	[139]
	Plantar pressure distribution	28	10^6^	CNN	Motion gait analysis	2024	[140]
DL for HMI	Stress on hand arrays	548	107	CNN	Recognition of grabbed items	2019	[55]
	Finger bending strain	5	15,000	CNN	Gesture recognition	2020	[141]
	Finger bending strain	5	10^6^	Transformer	Gesture recognition	2022	[142]
	Finger bending strain	10	10^5^	LSTM	Gesture recognition	2022	[143]
	Laryngeal movement	1	10^7^	CNN	Classification of voice and neck movements	2023	[144]
	Hand pressure and ethanol gas concentration	76	10^8^	CNN	Object recognition	2022	[145]
	Esophageal muscle movement	1	10^7^	CNN	Speech recognition	2023	[21]
	Oral muscle exercise	1	10^5^	RNN	Lip recognition	2021	[123]
	Speaking voice waveforms	1	10^6^	CNN	Speech recognition	2022	[146]
	Humidity, proximity, pressure	3	10^5^	LSTM	Object recognition	2023	[147]
	Laryngeal movement	1	10^6^	CNN	Speech recognition	2023	[148]
	Hand tactile information	2049	10^7^	CNN	Surface texture recognition	2021	[133]
	Lip muscle strain	8	10^7^	CNN	Speech recognition without voice	2022	[149]
	Acoustic oscillation	7	10^6^	CNN	Speaker identification	2022	[150]
	Facial muscle signals	2	10^5^	RNN	Emotion recognition	2024	[11]
	Muscle movement signals	1	10^5^	RNN	Classification of pronunciation	2024	[151]
	Facial muscle exercise	5	10^6^	CNN	Emotion recognition	2025	[152]
	Finger bending strain	5	10^5^	CNN	Gesture recognition	2024	[10]
	Wrist rotation	16	10^7^	CNN	Handwriting recognition	2023	[153]
	Temperature, pressure	9	10^6^	SNN	Object recognition	2022	[154]
	Modulus of elasticity	2	10^6^	CNN	Softness classification	2022	[20]
	Ammonia	60	10^5^	CNN	Food freshness monitoring	2020	[155]
	Hand tactile information	16	10^6^	CNN	Tactile mapping	2022	[16]
	Strain on different parts of the body	216	10^6^	CNN	Whole-body poses recognition	2021	[22]
	Ultrasound images of the heart	6	10^5^	CNN	Left-ventricular volume	2023	[156]
	Vocal dose	1	10^5^	CNN	Vocal fatigue	2023	[157]

## Data Availability

Data sharing is not applicable to this article.
